# Investigation of the Flame Retardant Properties of High-Strength Microcellular Flame Retardant/Polyurethane Composite Elastomers

**DOI:** 10.3390/polym14235055

**Published:** 2022-11-22

**Authors:** Xiaoxia Wu, Xudong Zhang, Jingpeng Wu, Xiaodong Li, Hao Jiang, Xing Su, Meishuai Zou

**Affiliations:** 1 School of Materials Science and Engineering, Beijing Institute of Technology, Beijing 100081, China; 2 School of Chemistry, Chemical Engineering and Materials Science, Zaozhuang University, Zaozhuang 277160, China; 3 Shandong AOZO New Materials Co., Ltd., Tengzhou 277524, China; 4 Lu’nan Research Institute of Beijing Institute of Technology, Tengzhou 277599, China

**Keywords:** microcellular polyurethane elastomer, flame retardant, expandable graphite, tensile strength

## Abstract

Flame retardants (FRs) often reduce the mechanical properties of polymer materials, and FR/microcellular polyurethane elastomer (MPUE) composite materials have not been systemically studied. Hence, we conducted this study on FR/MPUE composites by using multiple liquid FRs and/or expandable graphite (EG). Compared with liquid flame retardants, the LOI of an expandable graphite/dimethyl methylphosphonate (EG/DMMP) (3:1) combination was significantly increased (~36.1%), and the vertical combustion grade reached V-0 without a dripping phenomenon. However, the corresponding tensile strength was decreased by 17.5%. With the incorporation of EG alone, although the corresponding LOI was not a match with that of DMMP/EG, there was no droplet phenomenon. In addition, even with 15 wt% of EG, there was no significant decline in the tensile strength. Cone calorimeter test results showed that PHRR, THR, PSPR, and TSR were significantly reduced, compared to the neat MPUE, when the EG content surpassed 10 wt%. The combustion process became more stable and thus the fire risk was highly reduced. It was found that flame retardancy and mechanical properties could be well balanced by adding EG alone. Our proposed strategy for synthesizing FR/MPUE composites with excellent flame retardancy and mechanical properties was easy, effective, low-cost and universal, which could have great practical significance in expanding the potential application fields of MPUEs.

## 1. Introduction

Commercial microcellular polymer materials, also called polymer foams, possess great importance as barriers, lightweight boards, in energy conservation, as structural materials, etc. [1,2]. Therefore, high-performance microcellular polymer materials are widely utilized in a variety of applications such as large buildings, transportation, military equipment, precise instruments, etc. [1,3]. Among the existing microcellular polymer materials, polyurethane foams play a vital role in the above-mentioned applications because of their good mechanical strength, fatigue resistance, and anti-aging capability [1,3,4,5,6]. However, as they are composed of polymeric molecules, these organic materials are highly intrinsically flammable, which can cause severe catastrophes [3,5]. On the one hand, traditional polyurethane foams burn quickly and aggressively once being ignited [7,8], and this leads to the destruction of buildings and equipment, as well as loss of lives [9]. On the other hand, during combustion, huge amounts of heat and toxic substances are released, which further increase the risk of casualty [7]. Accordingly, the development of mechanically strong and flame retardant polyurethane foams has become important.

Great efforts have been made to achieve flame retardant polyurethane materials, including intrinsically flame retardant polyurethane, flame retardant coating, and flame retardant additives [5,7,10]. The introduction of flame retardant functional groups into polyurethane molecules may reduce the flammability of the polymer matrix [11]. However, it is difficult to balance the mechanical and flame retardant properties. Some components with considerable flammability are necessary for maintaining good mechanical properties, especially for microcellular polyurethane materials [5,7,11,12]. Besides, complex processing techniques and high costs are often involved in manufacturing intrinsically flame retardant polyurethane [3,5,7]. Facial coatings can enhance flame retardancy, and often have little impact on the chemical composition of the polyurethane matrix [3,5,13,14]. However, the coating layer is difficult to adaptwell to the polyurethane matrix upon cyclic stress [15,16,17]. This often leads to facial cracking, interfacial abscission, or even fracture [15,16,17]. As for microcellular polyurethane materials, mechanical adaptability between the coating layer and the porous polyurethane matrix could be even more challenging [18]. Flame retardant additives can be incorporated within the polyurethane matrix, forming composite materials [7,19]. Organic flame retardant additives include halogen, phosphorous, nitrogen, and silicon elements [20,21,22,23]. They can release elementary free radicals upon heating which efficiently capture flammable HO· and H· free radicals [24]. Inorganic additives include phosphorous additives, metal oxide/hydroxide, borate, silicate, and expandable flame retardant additives [25,26,27,28,29,30]. Among these, expandable graphite (EG), as a kind of expandable flame retardant additive, tends to expand upon heating, thus forming thick, porous, carbonized layers [19,28,31]. These layers possess high thermal stability, which can efficiently separate the polymer phases from the heat source, thus delaying and even terminating the decomposition of the polymer [19,28]. In addition, it displays no intrinsic toxicity and does not produce harmful gases when heated [19,26,28]. Moreover, it can greatly reduce the amount of smoke [19,26,28]. However, the incorporation of flame retardant additives usually significantly reduces the mechanical properties of the composite materials [32,33,34,35,36,37]. The current study on the flame retardancy of polyurethane focuses on rigid polyurethane foams, whose mechanical properties upon large deformation are not considered [38,39,40,41,42,43]. Some vital properties, including flame retardancy of newly developed microcellular polyurethane elastomers (MPUE), have not been systemically studied yet [11,31,44,45]. Unlike the rigid polyurethane foams, the mechanical properties of relatively soft MPUE are crucial in the applications of vibration attenuation, noise reduction, energy conservation, structural materials, etc. [1,2]. The flame retardant or other properties should be considered without compromising the mechanical properties.

Herein, we conducted research on the effect of liquid flame retardants including tri (2-chloroethyl) phosphate (TCEP), tri (2-chloropropyl) phosphate (TCPP), and dimethyl methylphosphonate (DMMP), as well as solid flame retardants including EG and EG/DMMP mixture, on the flame retardant properties of microcellular polyurethane composite elastomers. Their mechanisms of flame retardancy and the influence on mechanical properties were systematically investigated. It was found that, with the incorporation of EG (~15 wt%), the formation of molten droplets was significantly inhibited and the corresponding limiting oxygen index (LOI) was increased by 9.8%. At the same time, the tensile strength of microcellular polyurethane/expandable graphite composite elastomers did not decline in comparison to that of MPUE alone. These interesting phenomena were attributed to dense interfacial interactions via hydrogen and chemical bonding between EG and MPUE. Our research is of great value for the future design and application of microcellular polyurethane composite elastomers with high mechanical properties and security.

## 2. Experimental Methods

### 2.1. Materials

**Figure 1:**
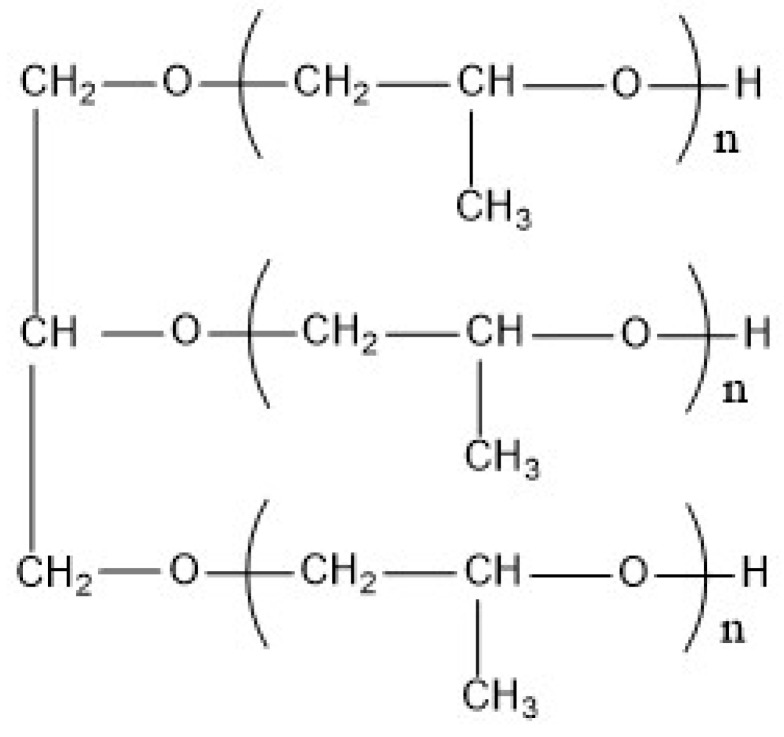
Chemical structure of polyether polyol 330N.

Polyether polyol 330N (Mn = 5000, f = 3, see Figure 1 for its detailed chemical structure) was supplied by Shandong Bluestar Dongda Co., Ltd., Zibo, China. Poly(tetrahydrofuran) (PTMEG1000, Mn = 1000, f = 2) and diphenylmethane diisocyanate (MDI) were purchased from BASF Co., Ltd., Ludwigshafen, Germany. Butanediol (BDO) was provided by Sichuan Tianhua Co., Ltd., Luzhou, China. Bis (dimethylaminoethyl) ether (BDMAEE) was bought from Yechuangxin Materials Co., Ltd., Shanghai, China. Amine catalyst A33 was supplied by Dongguan Guangsiyuan Polyurethane Material Co., Ltd., Dongguan, China. Foam homogenizing agent (BYK-9231) was purchased from Byk-Chemie GmbH, Wesel, Germany. Expandable graphite (EG) (granularity: 80 mesh, expansion rate: 300 mL/g) was bought from Qingdao Jintao Graphite Co., Ltd., Qingdao, China. Tri (2-chloropropyl) phosphate (TCPP), tri (2-chloroethyl) phosphate (TCEP), and dimethyl methyl phosphonate (DMMP) were bought from Shandong Baijiahe Chemical Technology Co., Ltd., Jinan, China. All of the chemicals were analytically pure and used as received. Table 1 shows the main physical and chemical properties of the main raw materials.

### 2.2. Synthesis of Microcellular Polyurethane/Flame Retardant Polyurethane Elastomers

Multiple chemicals including polyether polyol (330N, PTMEG1000), chain extender (BDO), foaming agent (water), polymerization catalyst (A33), foaming catalyst (BDMAEE), foam homogenizer (BYK-9231), flame retardant (FR) (EG, TCPP, TCEP, or DMMP) at specific stoichiometric ratios were mixed evenly via constant stirring, forming Component A (Table 1 shows more details about the composition of samples). Polyether polyol (PTMEG1000) and MDI were mixed at specific stoichiometric ratios, forming Prepolymer B. A and B (molar ratio of A:B = 1:1.02) were mixed at a specific temperature. After high-speed stirring, the mixture was poured into a flat mold and sealed for 30 min’ curing. The flat samples were then collected and subject to a ripening treatment at 70 °C for 24 h. Afterwards, the materials were placed at room temperature for 7 d to obtain the final microcellular polyurethane elastomer samples. The density of different samples was kept at about 500 kg/m^3^ by fine tuning the amount of foaming agent and raw materials. Standard samples were prepared according to relevant standards for various performance tests. A series of control samples were also synthesized, and the detailed composition is shown in Table 2 and Table 3.

### 2.3. Characterizations

The chemical structure of the materials was characterized by Fourier transform infrared spectroscopy (FTIR). Each sample was scanned during 8 cycles in a wavenumber range from 4000 to 500 cm^−1^ at the resolution of 2 cm^−1^, by using Thermo Nicolet is 50 equipment (Thermo Nicolet Corporation, Madison, Wisconsin, USA). The thermal stability of the materials was investigated via thermogravimetric analysis (TGA) under nitrogen atmosphere, by using Rigaku TG-DTA8122 equipment (Rigaku Corporation, Tokyo, Japan). The heating rate was set at 10 °C/min and the temperature range was within 25~800 °C. Limiting oxygen index tests (LOI) were conducted on a JF-3 LOI tester (Nanjing Jionglei Instrument Equipment Co., Ltd., Nanjing, China), following the standard of GB/T 2406.2-2009. The tested samples measured 150 mm × 10 mm × 10 mm. Vertical combustion tests were conducted on a CZF-5 vertical combustion tester (Nanjing Jionglei Instrument Equipment Co., Ltd.), following the standard of GB/T 2408-2021. The tested samples measured 125 mm × 13 mm × 3.0 mm. Cone calorimeter evaluation was conducted on a VOUCH6810 cone calorimeter (Suzhou Yangyi Volch Testing Technology Co., Ltd., Suzhou, China), following the standard of ISO 5660. The radiant power was set to 35 kW/m^2^. The tested samples measured 100 mm × 100 mm × 3.2 mm. Tensile tests were performed on a GDWEW-10 universal testing machine (Shanghai Genzhun Instrument & Equipment Co., Ltd., Shanghai, China), following the standard of GB/T 10654-2001. The thickness of the tested samples was 10 mm and a crosshead speed of 500 mm/min was used. Surface morphology of the materials was observed by scanning electron microscopy (SEM), by using an EM-30 PLUS high-resolution desktop scanning electron microscope (Coxem Co., Ltd., Daejeon, Republic of Korea). The accelerate voltage was set to 20 kV.

## 3. Results and Discussion

### 3.1. Chemical Structure of Flame Retardant (FR) and the Microcellular Polyurethane Composite Elastomers (FR/MPUE)

**Figure 2:**
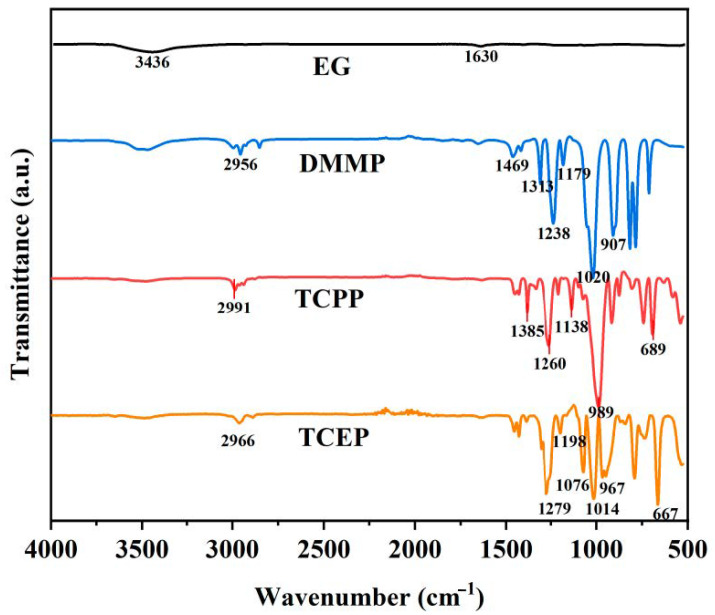
FTIR spectra of TCEP, TCPP, DMMP, and EG.

Figure 2 displays the characteristic chemical structures of the flame retardants used, including TCEP, TCPP, DMMP, and EG. For TCEP, the peaks at 2966 cm^−1^, 1279 cm^−1^, and 1198 cm^−1^ represented stretching vibration of CH_2_, P=O, and P^−^O^−^C, respectively [46]. The peaks at 1076 cm^−1^, 1014 cm^−1^, and 967 cm^−1^ corresponded to symmetric and asymmetrical stretching vibration absorption of the phosphate structure [47]. The peak at 667 cm^−1^ was due to C^−^Cl bonding. For TCPP, the peak at 2991 cm^−1^ represented CH_2_ and CH_3_ stretching vibration. The peak at 1385 cm^−1^ corresponded to isopropyl groups. The peaks at 1260 cm^−1^, 1138 cm^−1^, 989 cm^−1^, and 689 cm^−1^ corresponded to P=O, P^−^O^−^C, stretching vibration absorption of phosphate, and C^−^Cl, respectively [46,47]. DMMP displayed a peak of CH_3_ stretching vibration at 2956 cm^−1^, and peaks of P^−^C at 1469 cm^−1^ (stretching) or 1313 cm^−1^ (bending) [48]. The peaks at 1238 cm^−1^, 1179 cm^−1^, 1020 cm^−1^, and 907 cm^−1^ corresponded to P=O, C^−^O, P^−^O^−^C, and P^−^CH_3_ bonding [48,49,50,51]. For EG, the peaks at 3436 cm^−1^ and 1630 cm^−1^ corresponded to stretching and bending vibration of ^−^OH groups, respectively [43,44].

**Figure 3:**
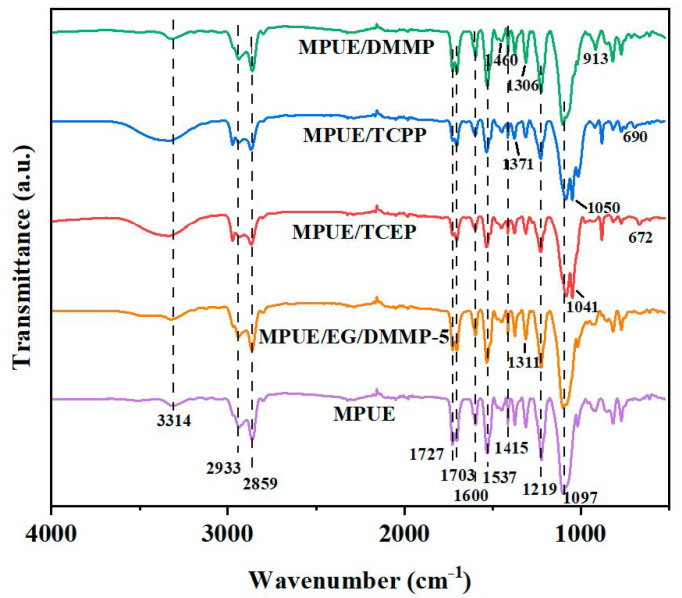
FTIR spectra of MPUE, MPUE/TCEP, MPUE/TCPP, MPUE/DMMP, and MPUE/EG/DMMP-5.

As depicted in Figure 3, the neat microcellular polyurethane elastomer (MPUE) showed peaks of N-H bonding in amide at 3314 cm^−1^ (stretching) and 1537 cm^−1^ (bending). The peaks at 2933 cm^−1^ and 2859 cm^−1^ were attributed to CH_2_ and CH_3_ stretching vibration, while the one at 1415 cm^−1^ represented the bending vibration of CH_2_ and CH_3_. The peaks at 1727 cm^−1^ and 1703 cm^−1^ indicated the free and hydrogen bonding restricted C=O groups, respectively. The peaks at 1602 cm^−1^, 1219 cm^−1^, and 1097 cm^−1^ corresponded to stretching vibration of the benzene backbone, C-O bonding in ester, and C-O-C bonding in polyether, respectively. Those characteristic peaks for polyurethane prove the formation of MPUE [44]. FTIR spectra also proved the formation of MPUE/flame retardant composite elastomers (FR/MPUE). MPUE/TCEP displayed peaks at 1041 cm^−1^ and 672 cm^−1^, which corresponded to phosphate and C-Cl. MPUE/TCPP displayed peaks at 1371 cm^−1^, 1050 cm^−1^, and 690 cm^−1^, which corresponded to isopropyl, phosphate, and C-Cl, respectively. MPUE/DMMP and MPUE/EG/DMMP-5 displayed peaks at 1460 cm^−1^, 1306 cm^−1^, and 913 cm^−1^, which corresponded to P-C and/or P-CH_3_. These phenomena indicated that those liquid flame retardants were physically dispersed in the MPUE matrix, without a participating chemical reaction. Noticeably, the absorption peak of -OH groups in EG shrank in MPUE/EG/DMMP-5, implying that the hydroxyl groups on EG took part in chemical crosslinking of MPUE/EG/DMMP. The main mechanism was attributed to the chemical reaction between hydroxyl and isocyanate groups, leading to carbamate bonding [6].

### 3.2. Effect of Liquid Flame Retardant on the Overall Properties of MPUE

The effect of incorporating TCEP, TCPP, or DMMP with the content of 15 wt% (in respect to the weight of the FR/MPUE) was studied and the results are listed in Table 4.

As shown in Table 4, the incorporation of any liquid flame retardant could effectively enhance LOI. Among them, DMMP displayed the best flame retardancy, which effectively inhibited the formation of molten droplets during burning. This could be attributed to the flame retardant mechanisms [5,12,45,51]. TCEP and TCPP tended to release PO· into the gaseous phase upon burning, which could capture the free radicals for chain growth of the combustion reaction [51]. On the other hand, apart from being a gaseous free radical capturer, DMMP could also build up a protective layer consisting of interpenetrating networks of carbon and phosphorus oxides, which were produced by thermal decomposition of DMMP. The protective layer effectively isolated the air and heat source [51].

Overall, the use of liquid flame retardant did not eliminate the phenomenon of dripping. This was due to the fact that, compared to traditional thermosetting polymers, the microstructure of microcellular polyurethane elastomers was prone to collapse upon heating and facilitated the formation of molten droplets [6,28,29]. Besides, the incorporation of liquid flame retardants significantly reduced the mechanical properties of MPUE composite materials. By incorporating a specific amount of liquid flame retardant, the tensile strength was reduced by over 37%, which is unacceptable for practical use. An explanation for this could be that the liquid flame retardants were of low molecular weight and viscosity, which did not attend chemical crosslinking of MPUE. Those unbounded small molecules significantly interfered the intermolecular forces of MPUE, displaying a prominent plasticizing effect and deteriorating the mechanical strength [18,23,38].

### 3.3. Effect of EG/DMMP Combination on the Overall Properties of MPUE

**Figure 4:**
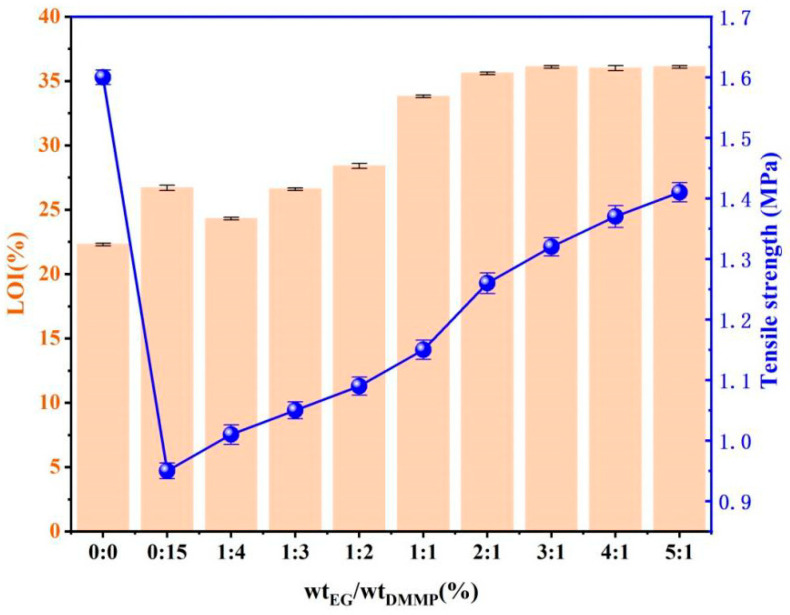
LOI and tensile strength of MPUE/EG/DMMP with different EG/DMMP ratios.

We considered the synergy of EG and DMMP on the flame retardancy of MPUE in this section. The total weight content of both EG and DMMP was 15% (in respect to the weight of the FR/MPUE). The varying ratios between them had a significant influence on the overall properties of MPUE. The corresponding results are shown in Table 5 and Figure 4.

**Figure 5:**
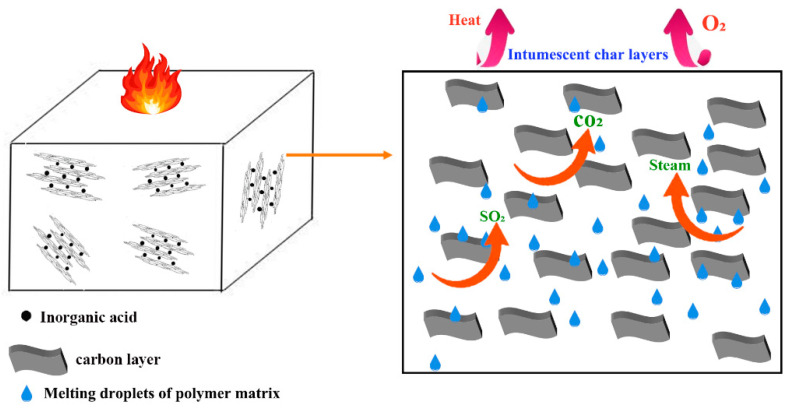
Schematic diagram of the flame retarding mechanism of EG.

LOI of MPUE initially went down and then went up with increasing EG content. When the EG/DMMP weight ratio reached 3, the maximum LOI value of 36.1% was obtained. As the EG/DMMP weight ratio reached 0.5, the vertical combustion grade reached V-0. Compared to DMMP alone, the EG/DMMP combination was beneficial for avoiding dripping and improving the mechanical strength of MPUE. The mechanisms can be explained by Figure 5. EG was a kind of layered graphite formed by the intercalative oxidation of natural flake graphite. Compounds (inorganic acids such as sulfuric acid, nitric acid, phosphoric acid, etc.) were introduced into the interlayer spacing. A redox reaction occurred upon heating, releasing large amounts of SO_2_, CO_2_, H_2_O, etc. Various gaseous substances could rapidly expand the interlayer spacing of EG and its volume was enlarged hundreds of times. The expanded carbon layers formed on the combustion surface played the important roles of oxygen insulation, heat insulation, and smoke suppression, creating good flame retardancy [28,29,30]. The incorporation of EG at a low level (EG/DMMP < 1/4) gave rise to lowered LOI. This was due to the fact that, upon heating, the low amount of EG in MPUE expanded drastically and the burning surface was significantly enhanced. This overwhelmed the flame retardant effect of the expanded graphite and thus reduced LOI. As the EG content went up further (>1/3), the expansion-induced flame retardant effect overcame the effect of the increased burning surface, leading to enhanced LOI. EG and DMMP showed a good synergistic effect. The reason was that DMMP was decomposed into a viscous byproduct, which covered the burning surface. This byproduct filled the void between the worm-like graphite layers, increasing densification. Therefore, dripping, as well as the transmission of oxygen or heat, was highly inhibited, resulting in increasing LOI [19,28].

As for the mechanical properties, the incorporation of EG tended to improve the tensile strength. On the one hand, there were abundant hydroxyl and ether groups on the surface of graphite, which could form hydrogen bonds with the MPUE matrix [19,42]. On the other hand, there were chemical bonds between EG and MPUE via carbamate chemistry. The synergistic physical and chemical interactions between MPUE polymer chains and EG platelets were suggested to enhance the stress transfer and the mechanical strength [52]. Overall, compared to the neat MPUE, the tensile strength of MPUE/EG/DMMP was reduced (>20%) because of the presence of DMMP.

### 3.4. Effect of EG on the Overall Properties of MPUE

**Figure 6:**
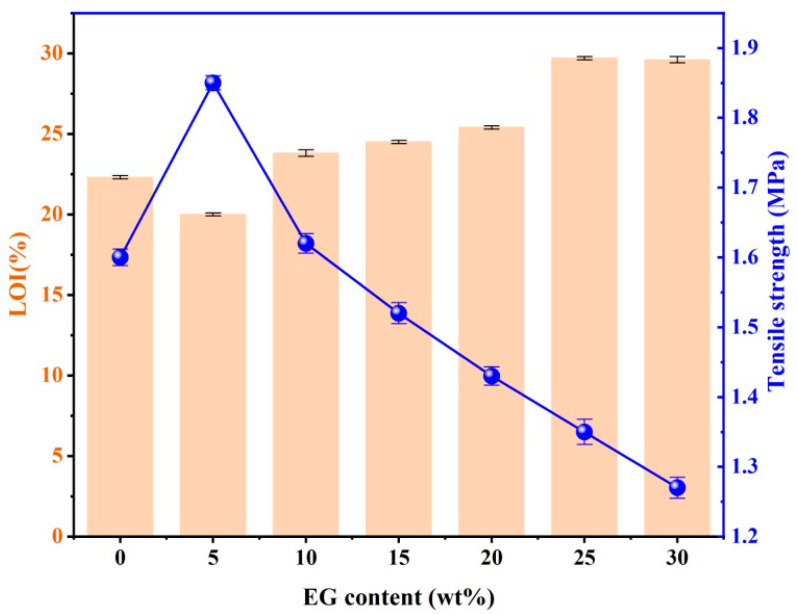
LOI and tensile strength of MPUE/EG at different EG contents.

As it was found that EG had an improving effect on the mechanical strength of MPUE, the effect of EG on the mechanical and flame retardant properties of MPUE was systemically studied, as shown in Table 6 and Figure 6.

With the incorporation of 5 wt% of EG, the corresponding LOI went down from 22.3% to 20%. Whereas, as the EG content kept increasing, LOI showed an increasing trend. This phenomenon could be explained by the fact that, at low levels of EG content (~5 wt%), the enhanced burning surface dominated, so that LOI declined. However, at high levels of EG content (>10 wt%), expansion-induced flame retardancy dominated so that LOI went up. This was different from the conclusion of a previous study [53], which indicated a strong flame retardant effect of EG on microcellular polyurethane elastomers and rigid porous polyurethane materials.

**Figure 7:**
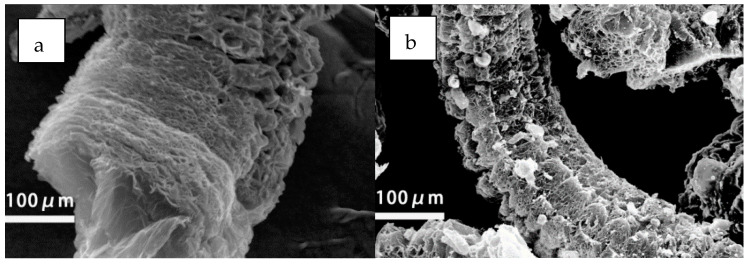
The residual carbon layers of (**a**) MPUE/EG and (**b**) MPUE/EG/DMMP.

Interestingly, when EG content was 15 wt%, there was no fallen carbon residue throughout the burning process. However, higher or lower EG content led to the apparent phenomenon of fallen carbon residue. This could be explained as follows. On the one hand, when EG content was low (<15 wt%), the expanded carbon layers were not able to effectively hold the molten droplets. In this case, carbon residue fell off with the molten droplets during the burning process. On the other hand, when EG content was high (>15 wt%), EG was not well dispersed in the MPUE matrix. Instead, some EG particles tended to stack together. Therefore, according to the “popcorn” effect [54], light carbon layers are prone to fall off upon combustion because of their weak interlayer interactions, causing fallen carbon residue. It was found that the appropriate amount of EG was vital for achieving ideal flame retardancy, and the influences of EG and EG/DMMP on dripping were different, which could be further explained by SEM. As shown in Figure 7, after burning, the residual carbon layers of EG were quite loose, while those of EG/DMMP were quite dense. This confirmed the formation of viscous interlayer substances by DMMP intercalated EG, as discussed before.

**Figure 8:**
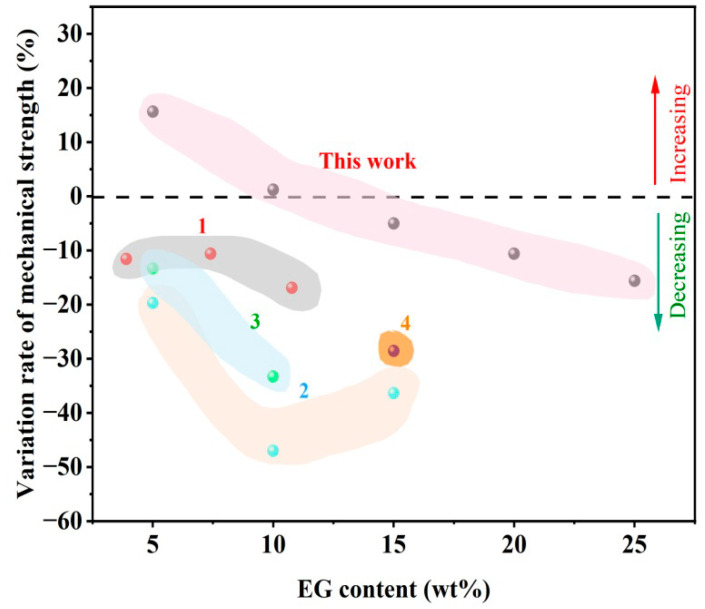
Effect of EG on the mechanical strength of polyurethane composite materials. The representative previous studies numbered 1 [38], 2 [55], 3 [40], 4 [41] of polyurethane foams are cited and compared here.

**Figure 9:**
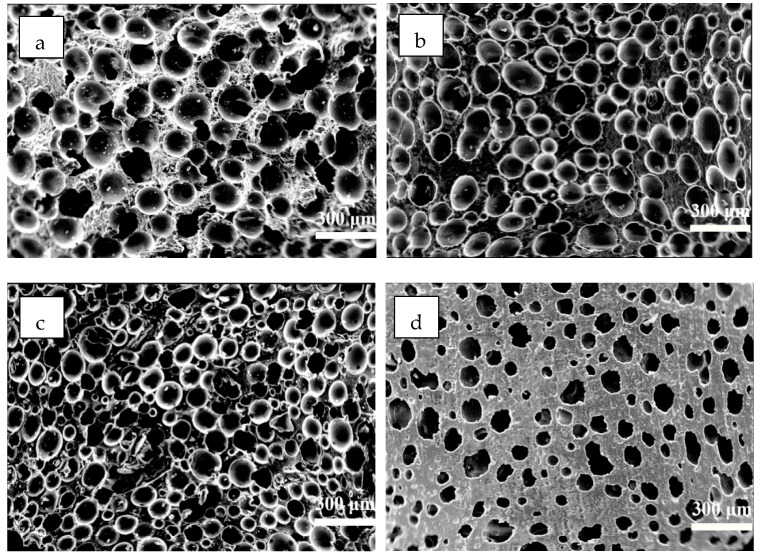
Cross-sectional area of MPUE/EG at the EG content of (**a**) 0 wt%, (**b**) 5 wt%, (**c**) 15 wt%, and (**d**) 30 wt%.

The incorporation of EG had an influence on the mechanical properties of MPUE/EG. Unlike previous studies showing reduced mechanical strength caused by incorporating FR (Figure 8), we found that by incorporating 5 wt% of EG, the corresponding tensile strength was increased by over 15%. Even when the EG content was increased to 15 wt% and 25 wt%, the corresponding tensile strength only declined by less than 5% and 15%, respectively. This high amount of EG displayed good flame retardancy as discussed before, comparable to that of MPUE/EG/DMMP at the same FR content (15 wt%). The mechanical strength of MPUE/EG in this work was also superior to that of MPUE incorporated with liquid flame retardant, as well as the previously reported EG incorporated rigid polyurethane foams (Figure 4, Figure 6 and Figure 8). The reason for the well-preserved mechanical strength was attributed to the fact that an appropriate amount of EG was well dispersed in the MPUE matrix. There was no apparent local stacking or EG platelet breaking out structure (Figure 9). Therefore, the incorporation of EG did not form large-scale phase separation or interruption on the microcellular structure, leading to well-preserved mechanical strength. However, once the EG content was further increased, the dispersion of EG became difficult and some EG platelets disrupted the microcellular structure, forming multiple defects in the form of opening pores. This could be confirmed by SEM (Figure 9). With the incorporation of 30 wt% of EG, the number of pores in MPUE/EG decreased and their dimensions became inhomogeneous, which is different from what happened with lower EG content. These factors significantly decreased the mechanical properties of MPUE/EG.

Under comprehensive consideration, when the EG content was 15 wt%~20 wt%, LOI was around 25%, and the corresponding tensile strength was reduced by only 5%~10.6%. Moreover, such EG content could effectively inhibit dripping and/or fallen carbon residue. Once 25 wt% of EG was introduced, despite a high LOI of 29.7%, the tensile strength was decreased by 15.6%. Therefore, MPUE/EG with 15 wt%~20 wt% of EG are ideal for these applications because of their good mechanical and flame retardant properties.

### 3.5. Thermal Stability of MPUE/EG

**Figure 10:**
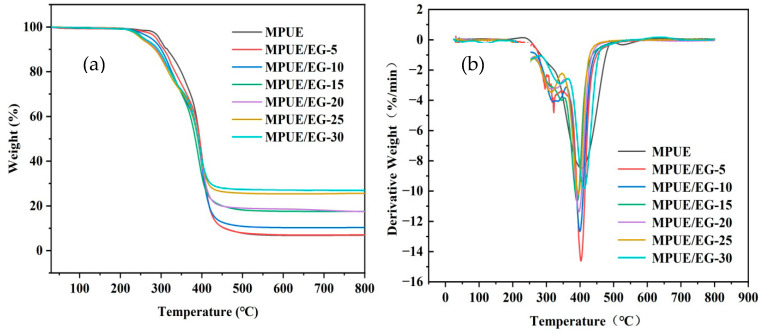
Thermogravimetric analysis (TGA) curves of MPUE/EG (**a**) TGA and (**b**) DTGA.

The thermal behaviors of MPUE and MPUE/EG were investigated through TG under nitrogen atmosphere. The corresponding results are presented in Figure 10 and Table 7. It was seen that the decomposition of the neat MPUE and MPUE/EG (5 wt% of EG) mainly consisted of two stages. The first one was between 285–350 °C, corresponding to the breakage of carbamate into diisocyanate and glycol [56]. The second one was between 350–500 °C, corresponding to the further decomposition of soft segment and hard segment [56]. When the EG content surpassed 10%, the initial decomposition temperature (T5%, temperature at 5% weight loss) decreased and the thermal decomposition process was promoted. There was an additional decomposition stage between 200–265 °C, which was mainly due to thermal decomposition of EG [57]. It should be noted that T_max_ (temperature at the maximum decomposition rate), corresponding to the extreme value of the thermal decomposition rate of all MPUEs, seldom shifted, and this was around 400 °C. However, the maximum mass loss rate (MMLR) decreased with increasing EG content, and the amount of solid residue increased with increasing EG content. Although the addition of EG hardly improved the initial thermal stability of MPUE, an adequate amount of EG could effectively retard the whole thermal decomposition process. This was due to highly expanded carbon layers upon heating, which efficiently isolated heat transmission.

### 3.6. Cone Calorimeter Analysis of MPUE/EG

The relevant data from the cone calorimetric testing are given in Table 8, including ignition time (TTI), heat release rate (HRR), peak heat release rate (PHRR), total heat release (THR), smoke release rate (SPR), peak smoke release rate (PSPR), total smoke release (TSR), time to reach the peak heat release rate (TTPHRR), carbon residue, etc.

**Figure 11:**
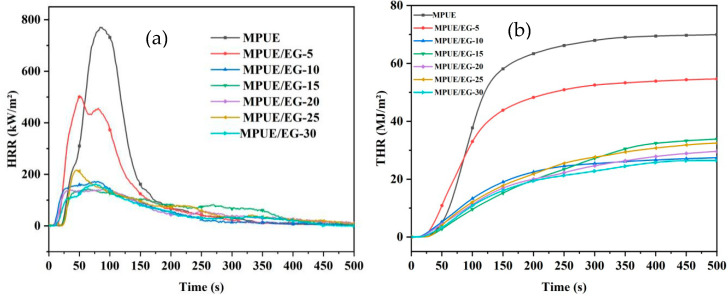
(**a**) HRR and (**b**) THR curves of MPUE/EG.

HRR, especially PHRR, is the most important parameter to characterize the fire intensity. It reflects the ability of flame to self-spread or spread to other flammable materials during combustion [28,29]. The greater the value, the more dangerous the fire is. It can be seen from Figure 11 that neat MPUE displayed a PHRR of 770.8 kW/m^2^ at 89 s. With the incorporation of 5 wt% of EG, the corresponding PHRR was reduced by 35% (501.5 kW/m^2^). When the EG content reached 10 wt%, PRHH was sharply decreased by 77% (172.8 kW/m^2^). With the higher EG content, the decrease of PHRR was not obvious. Compared to the neat MPUE, the TTI and TTPHRR of MPUE/EG were reduced, which was inconsistent with TGA results. The trend of the THR curves were the same as those of the HRR curves shown in Figure 11. When the EG content was 5 wt% and 10 wt%, THR was decreased by 21% and 60%, respectively. Further increasing EG content did not result in an obvious THR decrease. In summary, when incorporating over 10 wt% of EG in MPUE, the whole combustion process will be relatively stable and the fire risk will be effectively reduced. At the same time, we also found that further increasing the EG content had little effect on the combustion characteristics of MPUE/EG. This could explain why increasing only the EG content could increase LOI, but have little effect on the vertical combustion grade.

**Figure 12:**
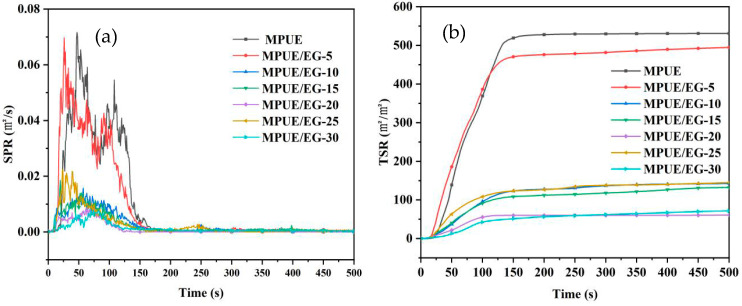
(**a**) SPR and (**b**) TSR curves of MPUE/EG.

As we know, smoke can be highly toxic and corrosive, and this cannot be ignored once a fire has started. Therefore, SPR and TSR were important indices for evaluating the combustion properties of materials. As shown in Figure 12, there were two peaks at 0.072 m^2^/s and 0.055 m^2^/s on the SPR curve of the neat MPUE, corresponding to two stages of its thermal decomposition. The SPR curve of MPUE incorporated with 5 wt% EG also displayed two peaks at 0.070 m^2^/s and 0.043 m^2^/s, which was similar to that of the neat MPUE. This indicates that a small amount of EG has no obvious effect of inhibiting smoke. When the EG content was 10 wt%, PSPR was 0.016 m^2^/s, which was only 22% of that of MPUE. There was only one peak at the SPR curve. This indicates that the introduction of EG greatly inhibited the generation of flue gas, and flue gas release was significantly slowed down during the whole combustion process. For higher EG content (>10 wt%), the trend of the SPR curve was basically consistent with that of MPUE/EG-10 (10 wt% of EG). However, the PSPR value did not significantly decrease with increasing EG content. Compared to the neat MPUE, the TSR of MPUE/EG-5 (5 wt% of EG) was decreased by only 10%. For MPUE/EG-10 (10 wt% of EG) and MPUE/EG-20 (20 wt% of EG), the corresponding TSR values were drastically decreased, by 75% and 89%, respectively.

The volume of EG can increase by 300 times in a moment when encountering high temperature, and the loose and porous worm shaped carbon layer generated can efficiently absorb and block the flue gas generated by the decomposition of the substrate. Besides, EG could effectively insulate oxygen and heat, retarding the combustion process. When the EG content was low, the few expanded carbon layers had a limited effect on the smoke suppression. When a larger amount of EG was introduced, TSR tended to increase. This was due to the fact that a high level of EG might produce smoke during combustion. In order to obtain a good smoke suppression effect, the EG content should be higher than 10 wt%. However, an EG content exceeding 20 wt% is not recommended because of the limited effect on suppressing smoke, and the reduced mechanical strength.

**Figure 13:**
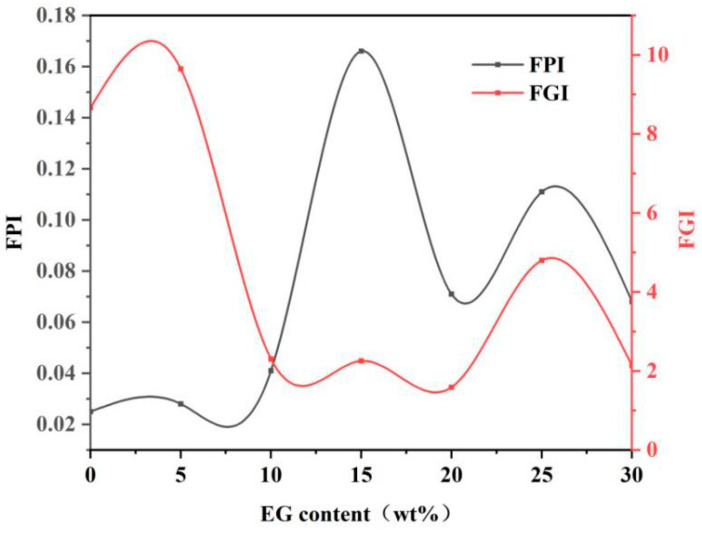
FPI and FGI curves of MPUE/EG.

In order to better describe the flame retardancy of materials, two factors including the fire performance index (FPI) and the fire growth index (FGI), can be mentioned [58]. FPI is defined as follows: FPI = TTI/PHRR. A large FPI corresponded to a low risk of the material catching fire. FGI is defined as the ratio of PHRR to the time for reaching PHRR. A large FGI corresponds to a high risk of the material catching fire. It can be seen from Figure 13 that MPUE/EG had a high FPI and a low FGI when the EG content was between 10 wt% and 20 wt%, indicating good fire safety.

**Figure 14:**
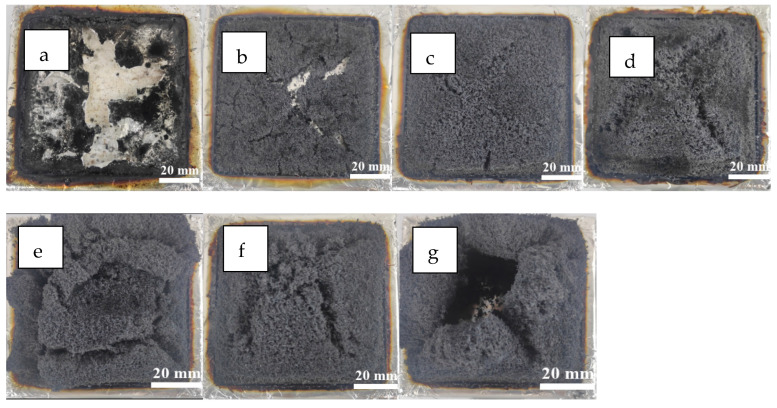
Digital photographs for the residues after cone calorimeter test: (**a**) MPUE, (**b**) MPUE/EG-5, (**c**) MPUE/EG-10, (**d**) MPUE/EG-15, (**e**) MPUE/EG-20, (**f**) MPUE/EG-25, and (**g**) MPUE/EG-30.

From the digital photos of polyurethane samples after CONE tests (Figure 14), it can be seen that there was seldom carbon residue on the neat MPUE sample (Figure 14a). There were large cracks and holes on the surface, indicating extensive heat release and smoke generation. The MPUE/EG samples had a lot of carbon residue. The loose microstructure was evenly distributed along the surface. These carbon layers played a vital role in heat insulation and smoke absorption. When the EG content was low, the carbon layer could not completely cover the combustion box. Only when the EG content exceeded 10 wt% could the expanded carbon layers completely cover the combustion box. However, if the EG content was too high, cracks or even large holes due to local collapse appeared on the surface. The binding force between the light expanded carbon layers and the thermal decomposition products of the matrix were fairly weak. With excessive EG, the greatly expanded carbon layers tended to “flow” under the impact of the flame. Therefore, cracks and local collapse occurred. This could also explain a finding of the vertical combustion test, that time taken for carbon residue to fall down was different for MPUE/EG at different levels of EG content.

## 4. Conclusions

In this paper, the effects of conventional flame retardants on the combustion and mechanical properties of MPUE were preliminarily studied. The LOI of FR/MPUE could be improved by adding 15 wt% of organic liquid flame retardants (TCEP, TCPP, or DMMP). However, dripping upon combustion and a significant decline in tensile strength (~37%) were inevitable. There was no dripping phenomenon for EG/DMMP/MPUE. However, the tensile strength of EG/DMMP/MPUE was still reduced (>20%) because of the presence of DMMP. EG alone had the potential to improve both flame retardance and mechanical properties. When the EG content surpassed 10 wt%, the corresponding PHRR, THR, PSPR, and TSR were reduced by 70%, 50%, 69%, and 75%, respectively. The appropriate EG content to achieve good mechanical and flame retardant properties was from 15 wt% to 20 wt%.

## Figures and Tables

**Table 1:** The main physical and chemical properties of the main raw materials.

Raw Material	Appearance (RT)	Hydroxyl Value (mgKOH/g)	NCO Mass Fraction (%)	Viscosity (25 °C, mpa s)	Water Content (wt, ppm)	Stability (NPT)	Flammability
330N	Colorless, transparent, and syrupy liquid	34	/	900	230	stable	flammable
PTMEG 1000	White, waxy, solid or colorless transparent liquid	112	/	3100	192	stable	flammable
BDO	Colorless, oily liquid	1247	/	72	215	stable	flammable
MDI	White solid	/	33.5	/	/	unstable	flammable

**Table 2:** The synthetic recipe of the neat MPUE.

Sample	MDI wt%	PTMEG1000 wt%	330 wt%	BDO wt%	H_2_O wt%	A33 wt%	BDMAEE wt%	BYK-9231 wt%
MPUE	23.5	39.4	33.3	3.2	0.14	0.01	0.13	0.32

**Table 3:** FR/MPUE composites studied in this work and their synthetic recipes.

Sample	Neat MPUE wt%	DCEP wt%	DCPP wt%	EG wt%	DMMP wt%	EG/DMMP wt%/wt%
MPUE/DCEP	85	15	0	0	0	/
MPUE/DCPP	85	0	15	0	0	/
MPUE/DMMP	85	0	0	0	15	/
MPUE/EG-5	95	0	0	5	0	/
MPUE/EG-10	90	0	0	10	0	/
MPUE/EG-15	85	0	0	15	0	/
MPUE/EG-20	80	0	0	20	0	/
MPUE/EG-25	75	0	0	25	0	/
MPUE/EG-30	70	0	0	30	0	/
MPUE/EG/DMMP-1	85	0	0	0	15	0:15
MPUE/EG/DMMP-2	85	0	0	3	12	1:4
MPUE/EG/DMMP-3	85	0	0	3.75	11.25	1:3
MPUE/EG/DMMP-4	85	0	0	5	10	1:2
MPUE/EG/DMMP-5	85	0	0	7.5	7.5	1:1
MPUE/EG/DMMP-6	85	0	0	10	5	2:1
MPUE/EG/DMMP-7	85	0	0	11.25	3.75	3:1
MPUE/EG/DMMP-8	85	0	0	12	3	4:1
MPUE/EG/DMMP-9	85	0	0	12.5	2.5	5:1

**Table 4:** Effect of liquid flame retardant on the overall properties of MPUE.

Sample	LOI %	Vertical Combustion Grade	Dripping	Tensile Strength MPa
MPUE	22.3 ± 0.1	NR *	Severe	1.60 ± 0.01
MPUE/TCEP	24.2 ± 0.1	NR	A little severe	0.91 ± 0.01
MPUE/TCPP	24.6 ± 0.1	V-2	A little severe	1.00 ± 0.02
MPUE/DMMP	26.0 ± 0.1	V-2	Slight	0.95 ± 0.01

* Please note that NR means the corresponding sample failed to pass the vertical combustion test.

**Table 5:** Effect of EG/DMMP with different ratios on the overall properties of MPUE.

Sample	EG/DMMP wt%/wt%	LOI %	Vertical Combustion Grade	Dripping	Tensile Strength MPa
MPUE	0:0	22.3 ± 0.1	NR	Severe	1.60 ± 0.01
MPUE/EG/DMMP-1	0:15	26.7 ± 0.2	V-2	Slight	0.95 ± 0.01
MPUE/EG/DMMP-2	1:4	24.3 ± 0.1	NR	None	1.01 ± 0.02
MPUE/EG/DMMP-3	1:3	26.6 ± 0.1	V-1	None	1.05 ± 0.01
MPUE/EG/DMMP-4	1:2	28.4 ± 0.2	V-0	None	1.09 ± 0.02
MPUE/EG/DMMP-5	1:1	33.8 ± 0.1	V-0	None	1.15 ± 0.02
MPUE/EG/DMMP-6	2:1	35.6 ± 0.1	V-0	None	1.26 ± 0.02
MPUE/EG/DMMP-7	3:1	36.1 ± 0.1	V-0	None	1.32 ± 0.01
MPUE/EG/DMMP-8	4:1	36.0 ± 0.2	V-0	None	1.37 ± 0.02
MPUE/EG/DMMP-9	5:1	36.1 ± 0.1	V-0	None	1.41 ± 0.02

**Table 6:** Effect of EG on the overall properties of MPUE.

Sample	EG wt%	LOI %	Vertical Combustion Grade	Dripping	Dripping Time (Start Timing after the Flame was Firstly Exerted.) s	Tensile Strength MPa
MPUE	0	22.3 ± 0.1	NR	Severe	Constant dripping	1.60 ± 0.01
MPUE/EG-5	5	20.0 ± 0.1	NR	Fallen carbon residue	7 s	1.85 ± 0.01
MPUE/EG-10	10	23.8 ± 0.2	NR	Fallen carbon residue	10 s	1.62 ± 0.01
MPUE/EG-15	15	24.5 ± 0.1	NR	No dripping or fallen carbon residue	None	1.52 ± 0.02
MPUE/EG-20	20	25.4 ± 0.1	NR	Fallen carbon residue	12 s	1.43 ± 0.01
MPUE/EG-25	25	29.7 ± 0.1	NR	Fallen carbon residue	8 s	1.35 ± 0.02
MPUE/EG-30	30	29.6 ± 0.2	NR	Fallen carbon residue	5 s	1.2 ± 0.01

**Table 7:** Thermogravimetry (TG) data of MPUE/EG.

Samples	T5% °C	T_max_ °C	MMLR %min^−1^	Char Residues at 700 °C %
MPUE	287	402	−14.6	7
MPUE/EG-5	286	402	−14.5	7
MPUE/EG-10	200	399	−12.7	10
MPUE/EG-15	258	391	−10.6	17
MPUE/EG-20	254	397	−11.4	17
MPUE/EG-25	250	395	−10.2	26
MPUE/EG-30	256	394	−10.4	27

**Table 8:** Cone calorimeter analysis of MPUE/EG.

Sample	TTI s	PHRR kW/m^2^	THR MJ/m^2^	PSPR m^2^/s	TSR m^2^/m^2^	Carbon Residue %	TTPHRR s	SF MW/m^2^	FPI	FGI
MPUE	19	770.8	70.3	0.072	529.3	3.2	89	408.0	0.025	8.661
MPUE/EG-5	14	501.5	55.0	0.070	480.1	8.3	52	240.8	0.028	9.644
MPUE/EG-10	7	172.8	27.8	0.016	129.9	24.6	75	22.4	0.041	2.304
MPUE/EG-15	24	144.4	34.8	0.014	113.7	31.9	64	16.4	0.166	2.256
MPUE/EG-20	10	141.2	30.7	0.009	58.9	27.0	89	8.3	0.071	1.587
MPUE/EG-25	24	216.0	33.0	0.022	127.4	30.9	45	27.5	0.111	4.800
MPUE/EG-30	11	162.1	27.00	0.008	66.3	32.4	76	10.7	0.068	2.133

## Data Availability

Not applicable.
